# Ethical Opinions in Research Ethics Committees: A Narrative Review of Normative Foundations, Deliberative Architecture, and Quality Standards

**DOI:** 10.1177/15562646261454440

**Published:** 2026-06-03

**Authors:** Helder Miguel R. O. Carreira

**Affiliations:** 1 56056 Department of Management and Economics, Universidade da Beira Interior, Covilhã, Portugal

**Keywords:** Research ethics committees, ethical opinion, deliberation, phronesis, bioethics, scientific integrity

## Abstract

Research ethics committees occupy a central position in scientific governance, yet the literature reveals a persistent gap in structured, theoretically grounded guidelines for drafting ethical opinions. Opinions are frequently characterised by insufficient substantiation and communicative opacity, eroding researcher trust and institutional legitimacy. This study provides an integrated framework for the production of quality ethical opinions. A narrative review was conducted through searches in PubMed, SCOPUS, and Web of Science, supplemented by normative documents from the WHO, Council for International Organizations of Medical Sciences, and World Medical Association. Opinion quality depends on three interdependent dimensions: normative grounding in foundational principles, deliberative integrity in collective processes, and communicative responsibility in documented reasoning. The most frequent deficiencies concern informed consent and risk-benefit analysis. Structured discretionary judgement proves superior to algorithmic standardisation. This work is original in integrating Habermasian communicative rationality with the Aristotelian concept of phronesis to propose a tripartite framework that transcends compliance-based approaches.

## Introduction

The ethical governance of research involving human participants constitutes one of the most consequential normative achievements in the history of modern science. Research ethics committees, designated Institutional Review Boards (IRBs) in North American jurisdictions and Research Ethics Committees (RECs) in European and international contexts, constitute the institutional infrastructure through which this governance is exercised. Their emergence is inseparable from the accumulated recognition of catastrophic violations of human dignity committed in the name of scientific progress: from the Nazi medical experimentation documented at the Nuremberg Trials, which generated the Nuremberg Code in 1947, to the Tuskegee Syphilis Study, whose exposure directly motivated the establishment of the National Commission for the Protection of Human Subjects of Biomedical and Behavioral Research (NCPHS) and the production of the Belmont Report (NCPHS, 1979). The Declaration of Helsinki (World Medical Association, 2024), now in its tenth revision, establishes prior independent ethical review as a mandatory condition for all medical research involving human subjects, a requirement since operationalised through national regulatory frameworks across more than 100 countries and through international guidelines including the Council for International Organizations of Medical Sciences (2016) standards.

Notwithstanding this institutional consolidation, a systematic and consequential gap persists in the literature: the absence of structured, theoretically grounded guidelines for drafting ethical opinions. The ethical opinion is the primary communicative output of REC deliberation: the document through which the committee's collective judgement is formalised, justified, and transmitted to researchers, institutions, and regulatory authorities. Its quality determines not only whether specific research participants will be adequately protected in individual studies, but whether the broader research community will perceive ethical review as a legitimate epistemic enterprise or as an arbitrary bureaucratic obstruction. Serpico (2024) demonstrates, through systematic analysis, that committees routinely fail to substantiate their requirements with corresponding ethical or regulatory justifications, a pattern that investigators legitimately experience as arbitrary gatekeeping and that erodes the institutional credibility of the entire review enterprise. Martín-Arribas et al. (2012), analysing the operational experience of a Spanish research ethics committee across a full evaluation cycle, found that approximately two-thirds of submitted protocols presented some form of ethical or administrative deficiency, confirming both the indispensability of structured review and the challenge of producing opinions that constructively orient researchers.

Previous research has addressed specific dimensions of REC governance, including composition and independence (Mehta et al., 2023), decisional consistency (Friesen et al., 2019), standards of evidence (Resnik, 2021), and informed consent processes (Capili & Anastasi, 2024), without integrating these dimensions into a unified framework oriented toward opinion quality. The normative foundations of research ethics review draw primarily from the principlist framework of Beauchamp and Childress (2019), organising ethical evaluation around prima facie principles of autonomy, non-maleficence, beneficence, and justice, and from the eight-requirement framework of Emanuel et al. (2004), which specifies the conditions that together render clinical research ethical, including the foundational requirement of collaborative partnership between researchers and host communities. These principlist approaches require supplementation by philosophical frameworks addressing the nature of collective judgement and the conditions under which principled ethical reasoning translates into transparent communicative action. The Aristotelian concept of *phronesis* (practical wisdom) (Aristotle, 2009) and Haberma'’s (1990) theory of communicative action provide precisely this supplementation, situating the ethical opinion as an act of reasoned practical judgement that is simultaneously philosophical, institutional, and communicative.

This study is guided by three research questions. First: what normative foundations and deliberative conditions are required for ethically sound and communicatively responsible REC opinions? Second: what structural and communicative standards determine the quality of ethical opinions? Third: what institutional conditions, including training, operating procedures, and feedback mechanisms, support the consistent production of quality opinions? The general objective is to provide an integrated conceptual and operational framework for the production of quality ethical opinions. The specific objectives are: (a) to map the normative foundations governing ethical evaluation in research contexts; (b) to analyse the deliberative conditions that enable the collective construction of sound ethical judgements; (c) to identify the structural and communicative standards that determine opinion quality; and (d) to examine the institutional conditions that support sustained quality in ethical review practice. The relevance of this study is simultaneously theoretical, advancing the integration of principlist, phronetic, and communicative frameworks; practical, providing actionable guidelines for committee members and governance administrators; and social, contributing to a research culture in which ethical review is perceived as a legitimate and pedagogically productive enterprise. This article is organised as follows: Section 2 maps the relevant literature; Section 3 describes the methodology; Section 4 presents findings integrated with the tables and figure; Section 5 discusses findings in relation to existing literature; Section 6 concludes with theoretical and practical implications, educational implications, best practices, a research agenda, and final considerations.

## Literature Review

### Normative Architecture: Principles, Rights, and Their Hermeneutic Application

The normative architecture governing research ethics is built upon a succession of foundational documents that collectively constitute the moral grammar of the international research community. A growing body of work suggests that these documents are not merely historical artefacts but active normative frameworks whose application to specific research contexts requires ongoing interpretive work. The Belmont Report (NCPHS, 1979) organised the normative foundations of human subjects research around three principles, namely respect for persons, beneficence, and justice, that have become the foundational reference for regulatory frameworks worldwide. Respect for persons requires that individuals be treated as autonomous agents and that those with diminished autonomy receive adequate protection. Beneficence encompasses two complementary obligations: not to inflict harm, and to maximise possible benefits while minimising foreseeable risks. Justice requires equitable distribution of the burdens and benefits of research, interrogating the power asymmetries that determine who participates, who bears risks, and who benefits from advances in knowledge.

Beauchamp and Childress (2019), in the most systematic treatment of biomedical ethics principles available in the contemporary literature, extend the Belmont framework to four principles, adding non-maleficence, and characterise them as prima facie obligations rather than absolute rules. This characterisation is theoretically decisive: if principles are prima facie rather than absolute, their application to specific cases requires judgement about relative weight in situations of genuine conflict. No algorithm can fully determine this judgement in advance. Emanuel et al. (2004) propose a complementary eight-requirement framework specifying the conditions that together render clinical research ethical: collaborative partnership; social or scientific value; scientific validity; fair subject selection; favourable risk-benefit ratio; independent review; informed consent; and respect for enrolled subjects. This framework integrates normative principles with their operational conditions, providing a structured evaluative instrument that committees can apply systematically. Crucially, independent review is identified as constitutive of ethical research rather than as a supervenient administrative requirement, establishing the REC function as intrinsic to research integrity. The addition of collaborative partnership as the eighth requirement explicitly addresses the relational obligations of researchers toward host communities, a dimension of particular importance in cross-cultural and international research contexts.

Brothers et al. (2019) argue persuasively that the Belmont framework, while conceptually robust, requires substantive extension to address twenty-first-century challenges including digital research, big data, and population-level interventions generating novel risk profiles not anticipated in 1979. The Council for International Organizations of Medical Sciences (2016) international guidelines, in their most recent revision, represent the most comprehensive operationalisation of research ethics principles for international contexts, addressing fair benefit sharing, community engagement, and the special obligations of researchers working with vulnerable populations in low- and middle-income countries. By contrast with earlier studies focused exclusively on Western institutional contexts, the Council for International Organizations of Medical Sciences (2016) revision extends the scope of ethical review to encompass structural inequalities and global health justice dimensions that the Belmont framework addresses only implicitly. Resnik (2021) formalises the irreducibility of ethical judgement by demonstrating that IRB decisions necessarily incorporate a value component that cannot be derived from empirical evidence alone. This finding has direct implications for committee practice: the quality of ethical review depends on the quality of the practical reasoning through which principles are applied, not on the comprehensiveness of the rule-base consulted.

### Institutional Architecture and Conditions of Deliberative Legitimacy

Research ethics committees are constituted as independent bodies whose legitimacy derives from their structural independence from the institutions whose research they review. The European Patients’ Academy on Therapeutic Innovation (European Patients’ Academy on Therapeutic Innovation, 2021) stresses that this independence is not a procedural formality but the condition under which participant interests can consistently be prioritised over those of sponsors, investigators, and institutions. Mehta et al. (2023), in the most comprehensive recent empirical study of REC governance, examining 146 national ethics committees, which document that 82.7% include a philosopher or specialist in bioethics among their members. This proportion reflects the recognition that the questions RECs address are irreducibly philosophical, requiring competencies that surpass the methodological scope of any single empirical discipline. The WHO (2011) operational standards specify that committee composition should be multidisciplinary and multiprofessional, including scientific and non-scientific members, legal expertise, and patient or public representatives where appropriate.

Convergent evidence indicates that compositional diversity is necessary but insufficient: without adequate training, appropriate standard operating procedures (SOPs), and a deliberative culture that genuinely values heterogeneous perspectives, diverse committees may default to deference toward the most scientifically credentialled members, negating the deliberative benefits of compositional variety. Standard operating procedures governing quorum requirements, conflict of interest management, documentation practices, and conditions for external consultation provide the structural framework within which genuine deliberation becomes possible. In contrast with earlier studies focused on compositional requirements alone, Mehta et al. (2023) draw attention to significant variation in committee capacity across world regions, noting that limited resources, inadequate training, and weak regulatory frameworks constrain ethical review quality in precisely those contexts where participant vulnerability may be greatest.

### Deliberative Processes: Communicative Rationality and the Construction of Collective Judgement

Ethical deliberation constitutes the substantive core of REC work and cannot be understood adequately through a proceduralist lens alone. Drawing on Haberma'’s (1990) theory of communicative action, deliberation may be defined as a communicative praxis in which participants orient themselves toward mutual understanding through the force of the better argument, rather than toward the strategic achievement of predetermined outcomes. Three conditions are necessary for deliberation to satisfy this communicative ideal: sincerity, requiring that participants genuinely express their considered views; inclusivity, requiring that all relevant perspectives have access to the deliberative space; and the authority of argument, requiring that positions be revised in response to compelling reasons rather than social pressure or institutional hierarchy. The European Patients’ Academy on Therapeutic Innovation (2021) guidelines operationalise these conditions procedurally: documentation must be distributed and studied before sessions; all members must have adequate time to contribute; and consensus must take priority over voting except in exceptional circumstances.

Habermas's theoretical architecture extends, however, beyond the procedural conditions of communicative action to encompass a broader democratic framework whose implications for research ethics governance remain underexplored in the existing literature. The three pillars of his political philosophy – the public sphere, communicative action, and deliberative democracy – are mutually constitutive: the public sphere designates the institutional space in which communicative action becomes politically effective, and deliberative democracy specifies the normative requirements that legitimate collective decision-making must satisfy in pluralist societies (Habermas, 1990). Research ethics committees, understood within this expanded framework, are not merely procedural filters for individual research protocols but nodes in the democratic governance of science: institutional spaces in which the public's stake in the ethical conduct of research is exercised through structured deliberation. This democratic function implies obligations that proceduralist accounts of REC governance do not fully capture, including transparency toward the public, accountability to broader social values, and responsiveness to the concerns of communities affected by research. Integrating research ethics governance within the concept of deliberative democracy, as distinct from the narrower concept of communicative action, would constitute a productive extension of the theoretical framework presented in this study.

Friesen et al. (2019) articulate the central theoretical tension in REC decision-making as a dilemma between algorithmic and discretionary judgement. An algorithmic system achieves consistency at the cost of sensitivity to morally relevant contextual particulars; a discretionary system achieves sensitivity at the cost of predictability. Drawing on the analogy with criminal sentencing systems, Friesen et al. (2019) argue for structured discretionary decision-making that provides explicit normative guidance while preserving the deliberative space required for phronetic judgement. This position aligns with Resnik's (2021) demonstration that REC decisions necessarily incorporate normative judgements irreducible to empirical evidence, and with Aristotle's (2009, Book VI) account of phronesis as the capacity to discern the appropriate course of action in situations where general principles underdetermine the particular case. Markham and Buchanan (2012) reinforce this analysis by identifying the grey areas characteristic of applied ethics as requiring enhanced deliberative practice rather than procedural resolution.

### The Ethical Opinion: Structure, Communicative Function, and Quality Determinants

The ethical opinion performs multiple functions simultaneously: it is a legal instrument authorising, conditioning, or refusing research activity; a philosophical argument articulating the normative reasoning behind a collective judgement; a communicative act transmitting that reasoning to investigators and institutions; and a pedagogical resource contributing to the ethical formation of the research community. An emerging body of work suggests that these functions are co-constitutive: an opinion that satisfies its legal function while failing its communicative function undermines the pedagogical legitimacy of the review enterprise as a whole. Serpico (2024), in the most systematic empirical analysis of IRB application quality available in the literature, identifies transparency as the foundational communicative obligation of ethical opinion drafting: committees that issue requirements without corresponding normative justifications produce what investigators legitimately experience as arbitrary institutional power. Chirico and Bramstedt (2022) propose an expanded vision of the committee's communicative role, arguing that RECs should serve as deliberative forums connecting scientists, editors, and policymakers, recognising that ethical governance extends beyond the pre-approval phase and that committees with genuine deliberative capacity are institutional resources for the broader scientific community. Collectively, the literature implies that the quality of the written opinion is both a condition and an expression of the quality of the deliberation it records.

## Methods

### Research Design and Methodological Justification

A narrative review was conducted to map, synthesise, and critically evaluate the theoretical and empirical literature on the structure, processes, and quality standards of ethical opinions produced by RECs. The narrative approach was selected in preference to systematic review for three reasons. First, the research questions require the integration of philosophical and empirical literatures that employ incommensurable methodologies and cannot be evaluated through a common quality appraisal framework. Second, the objective is the construction of an integrative analytical framework rather than the statistical aggregation of effect estimates. Third, the theoretical traditions relevant to the analysis, namely moral philosophy, bioethics, institutional theory, and organisational behaviour, operate through argumentative logic rather than the hypothesis-testing paradigm that systematic review presupposes. The methodological transparency concerns characteristic of systematic reviews were addressed through explicit documentation of search strategy, inclusion and exclusion criteria, and analytical procedures.

### Search Strategy

Searches were conducted in PubMed, SCOPUS, and Web of Science during October–November 2024. The following terms were employed in varied combinations using Boolean operators: research ethics committee; institutional review board; ethical opinion; IRB decision; bioethics deliberation; phronesis research ethics; informed consent review; committee consistency; ethics review quality; REC governance; and ethical deliberation. No lower date boundary was applied; an upper boundary of November 2024 was used. Searches were conducted without language restriction; the synthesis incorporates primarily English, Portuguese, and Spanish sources, reflecting the authors’ linguistic competences and the geographic distribution of available literature. Reference lists of retrieved articles were screened systematically for additional relevant works. Normative documents from the WHO (2011), Council for International Organizations of Medical Sciences (2016), and World Medical Association (2024) were incorporated as primary regulatory references.

### Inclusion and Exclusion Criteria

Inclusion criteria were: peer-reviewed theoretical or empirical studies addressing REC or IRB structure, composition, deliberative processes, opinion drafting, decision-making consistency, or member training; normative guidelines from internationally recognised bodies; and philosophical analyses of ethical deliberation with institutional relevance for RECs. Exclusion criteria were: studies addressing ethics review exclusively within narrow technical sub-disciplines without implications for general committee practice; grey literature from non-recognised sources without traceable institutional provenance; and commentary articles without reference to primary empirical or theoretical sources.

### Data Extraction and Thematic Synthesis

Data extraction involved the systematic annotation of each included source for: theoretical framework employed; dimensions of REC practice or opinion quality addressed; principal arguments or empirical findings; methodological approach for empirical studies; limitations acknowledged; and relevance to the three research questions. Extracted data were organised into a thematic synthesis matrix structured around four areas: normative foundations; institutional architecture; deliberative processes; and documentary standards. The synthesis privileged the construction of an integrated analytical argument over the sequential summarisation of individual sources, identifying points of convergence, divergence, and theoretical complementarity across sources.

### Ethical Considerations

This study involves exclusively the analysis of published, publicly available academic literature and normative documents and does not involve primary data collection from human participants. No research ethics committee (REC) approval was required. All sources are cited accurately in accordance with the intellectual property conventions applicable to academic publication. The limitations of available evidence, including its geographic concentration and the absence of validated quality assessment instruments, are disclosed fully in the discussion.

## Results

**Figure 1.**
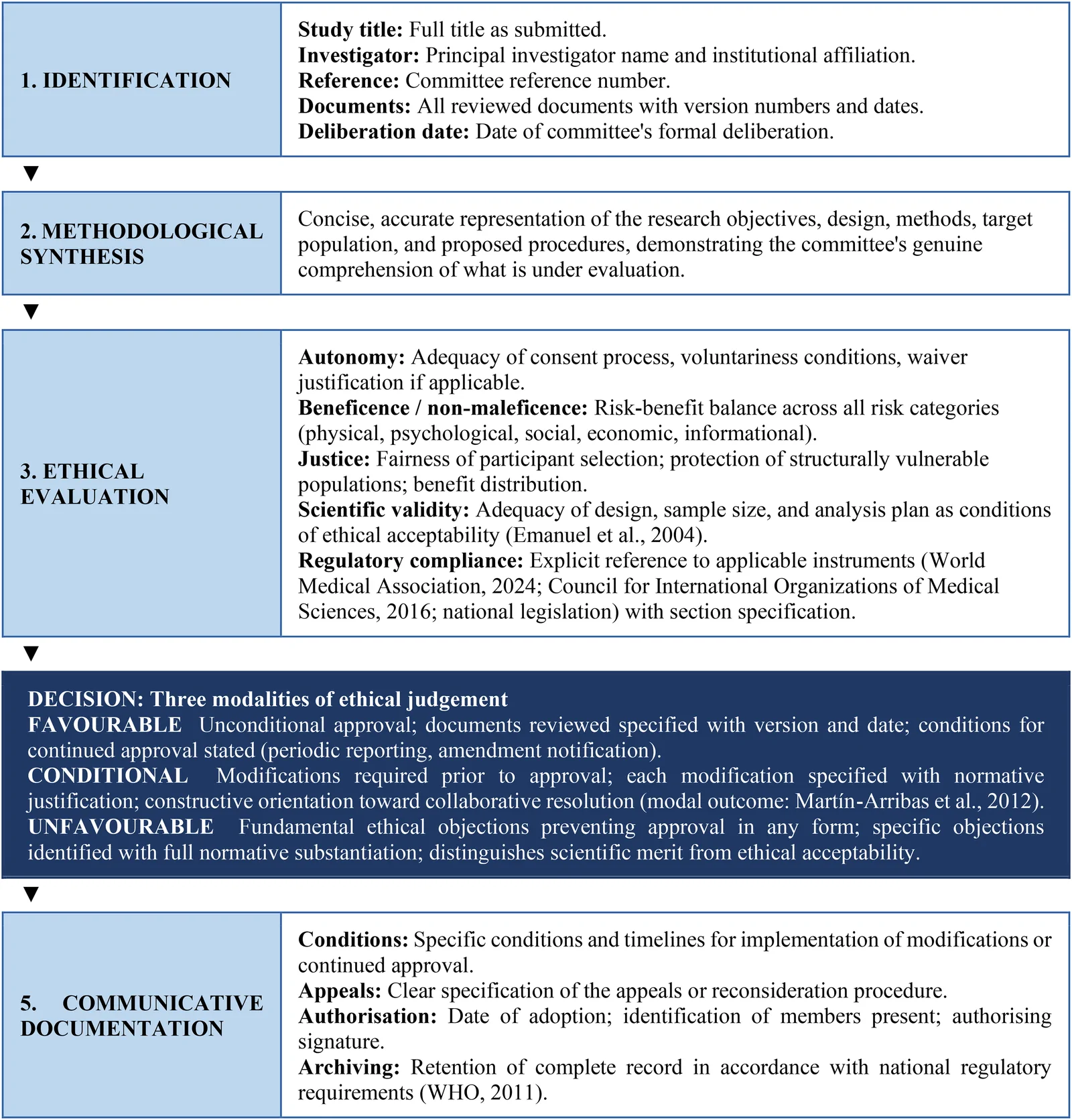
Structural architecture of a quality ethical opinion.

*Note:* The five-stage architecture represents the logical progression from identification through systematic ethical evaluation to communicative documentation. The decision stage encompasses three modalities: favourable (unconditional approval), conditional (modifications required), and unfavourable (fundamental ethical objections). Each stage is necessary; no stage substitutes for another. Sources: WHO (2011); *World Medical Association (2024)*; *Council for International Organizations of Medical Sciences (2016)*; *Emanuel* et al. *(2004)*; *Serpico (2024)*; *Martín-Arribas* et al. *(2012)*.

The thematic synthesis produced four interconnected findings. Table 1 provides a comparative synthesis of the principal normative frameworks and their operational implications for opinion drafting. Table 2 presents the tripartite framework for opinion quality across its three dimensions. Figure 1 illustrates the structural architecture of a quality ethical opinion.

**Table 1.** Comparative Synthesis of Principal Normative Frameworks and Their Implications for Ethical Opinion Drafting.

Framework	Core Principles / Requirements	Operational Implications for Opinion Drafting	Principal Limitations
Belmont Report (NCPHS, 1979)	Respect for persons; beneficence; justice	Opinions must address autonomy (consent process), risk-benefit balance, and equitable participant selection as distinct evaluative dimensions, each substantiated with reference to its corresponding principle.	Does not address digital or population-level research risks; concentrated in North American regulatory context; requires extension for twenty-first-century challenges (Brothers et al., 2019).
Beauchamp & Childress (2019)	Autonomy; non-maleficence; beneficence; justice as prima facie obligations (not absolute rules).	Opinions must articulate how competing prima facie principles were weighted in the specific case; evaluative conclusions cannot be presented as automatic derivations from stated principles.	Individual-level framework requires adaptation for collective institutional application; does not specify the deliberative conditions for committee practice.
Emanuel et al. (2004)	Eight requirements: collaborative partnership; social/scientific value; scientific validity; fair subject selection; favourable risk-benefit ratio; independent review; informed consent; and respect for subjects.	Provides a structured eight-point evaluative instrument for opinion writing, extending the original framework to developing-country contexts and adding collaborative partnership as an eighth requirement. Identifies independent review as constitutive of ethical research. Opinions should address each requirement explicitly, including collaborative partnership obligations.	Developed primarily for clinical research in developing countries; extension to qualitative, observational, and digital research requires interpretive work. The collaborative partnership requirement, while essential in cross-cultural contexts, may require adaptation for single-country studies.
Council for International Organizations of Medical Sciences (2016)	All Belmont principles extended internationally; fair benefit sharing; community engagement; special obligations in LMICs; protection of structurally vulnerable populations.	Opinions involving international collaborations or vulnerable populations must address structural inequalities, benefit distribution, and community engagement processes beyond individual participant consent.	Limited uptake in national regulatory frameworks outside Europe and North America; tension with national sovereignty; implementation capacity varies significantly across world regions.
World Medical Association Declaration of Helsinki (2024)	Mandatory prior independent ethical review; protection of research subjects; transparency in publication; post-trial access to proven interventions.	Prior review requirement mandates opinions be issued before research commencement. Post-trial access obligations extend committee responsibility beyond the approval phase to outcomes monitoring.	Focus on medical research; application to social, behavioural, and digital research requires contextual adaptation not specified in the Declaration.

*Note:* LMICs = low- and middle-income countries. NCPHS = National Commission for the Protection of Human Subjects of Biomedical and Behavioral Research.

**Table 2.** Tripartite Framework for Ethical Opinion Quality: Dimensions, Indicators, and Deficiencies.

Dimension	Definition	Quality Indicators	Common Deficiencies
Dimension I: Normative grounding			
Principlist foundation	Rigorous application of foundational ethical principles as prima facie obligations requiring judgement, not mechanical rule-following.	Each evaluative conclusion references a specific principle (autonomy, beneficence, justice, non-maleficence) or normative requirement (Emanuel et al., 2004). Competing principles are explicitly weighed.	Opinions that cite principles in the preamble but do not connect them to specific evaluative conclusions. Generic references to ‘ethical standards’ without specification.
Regulatory grounding	Explicit connection of each requirement or observation to the applicable regulatory provision or guideline.	Each observation specifies the regulatory instrument invoked (e.g., World Medical Association Declaration of Helsinki, Council for International Organizations of Medical Sciences 2016, national legislation) with section or article reference.	Requirements issued without regulatory citation. Researchers unable to identify the normative source of committee demands, perceiving review as arbitrary (Serpico, 2024).
Contextual sensitivity	Recognition of morally relevant particulars that distinguish the case under review from general categories addressed by principles and regulations.	Opinion addresses specific features of the protocol, including population vulnerability, research setting, and risk profile, that require contextual application of general principles (Aristotle, 2009; Resnik, 2021).	Formulaic opinions that apply identical language across diverse protocols. Failure to engage with contextually specific ethical concerns (Friesen et al., 2019).
Dimension II: Deliberative integrity			
Communicative conditions	The deliberative process satisfies Habermasian norms of sincerity, inclusivity, and the authority of argument (Habermas, 1990).	Documentation distributed before meetings; all members contribute; positions revised in response to argument not authority; consensus prioritised over voting (European Patients’ Academy on Therapeutic Innovation, 2021).	Deliberations dominated by most senior or scientifically credentialled members. Premature convergence. Voting substituted for deliberation in non-exceptional circumstances.
Structured discretion	Judgement that is contextually sensitive and consistently principled: neither algorithmically rigid nor arbitrarily variable (Friesen et al., 2019).	Deliberative reasoning documented with sufficient specificity to distinguish principled from arbitrary variation across cases. Retrospective review of decision consistency.	Inconsistent decisions on comparable protocols without documented normative justification for differences. Either algorithmic rigidity (excluding morally relevant particulars) or unconstrained arbitrariness.
Collective phronesis	Practical wisdom as an institutional capacity cultivated through sustained deliberative experience, reflection, and learning (Aristotle, 2009).	Committee engages in structured reflection on difficult cases; exchanges practice with peer committees; develops institutional memory through documented precedent.	Absence of mechanisms for institutional learning. High member turnover without knowledge transfer. No structured reflection on past decisions.
Dimension III: Communicative responsibility			
Transparency	Full disclosure of the reasoning through which the committee's judgement was constructed, enabling rational scrutiny by researchers and regulatory authorities.	Every requirement or observation accompanied by normative justification. Absence of opaque or undifferentiated demands. Researchers can identify and address specific concerns (Serpico, 2024).	Requirements unaccompanied by justification. Researchers experience review as arbitrary gatekeeping. Opacity erodes institutional legitimacy of the committee's function.
Specificity and actionability	Conditional opinions specify required modifications with sufficient precision to enable focused, targeted responses from research teams.	Modifications specified with reference to the section of the protocol requiring change, the normative basis for the requirement, and the standard against which revised submissions will be assessed.	Vague requirements (e.g., ‘improve the consent process’) that do not identify what specifically must change or why. Multiple iterations caused by imprecise initial opinions (Martín-Arribas et al., 2012).
Pedagogical function	Opinion functions as a contribution to the ethical formation of the research team, not merely as an administrative determination.	Explanatory reasoning connects specific protocol features to ethical principles in ways that build researcher competence. Constructive orientation toward resolution rather than compliance per se.	Opinions that communicate conclusions without explanation. Purely administrative tone that forecloses ethical dialogue between committee and research community (Chirico & Bramstedt, 2022).

*Note:* REC = research ethics committee. SOPs = standard operating procedures. The three dimensions of normative grounding, deliberative integrity, and communicative responsibility are interdependent and cannot be achieved independently of one another.

### Theme 1: The Irreducibility of Normative Foundations to Procedural Compliance

A convergent finding across the reviewed literature is that the normative foundations of ethical opinion, namely the principlist framework of Beauchamp and Childress (2019), the eight requirements of Emanuel et al. (2004), and the international guidelines of Council for International Organizations of Medical Sciences (2016) and World Medical Association (2024), do not constitute a procedural checklist but an interpretive framework whose application to specific cases requires practical reasoning qualitatively different from rule-following. The prima facie character of ethical principles (Beauchamp & Childress, 2019) implies that ethical review involves the exercise of judgement about relative normative weight in situations of genuine conflict. Resnik (2021) formalises this irreducibility by demonstrating that IRB decisions necessarily incorporate a value component that cannot be derived from empirical evidence alone. Brothers et al. (2019) extend this analysis to twenty-first-century contexts, demonstrating that the Belmont framework requires substantive extension to address digital risks and population-level interventions, confirming that the normative foundations of ethical review are not a static heritage but a living framework requiring ongoing reflexive development. Table 1 presents a comparative synthesis of the five principal normative frameworks with their core principles, operational implications for opinion drafting, and principal limitations.

### Theme 2: Deliberative Conditions as Primary Determinants of Opinion Quality

The reviewed literature consistently identifies deliberative integrity, understood as the quality of the collective reasoning process through which the committee constructs its judgement, as the primary determinant of opinion quality. Friesen et al. (2019), in the most systematic theoretical treatment of REC decision-making in the literature, demonstrate that the central challenge is not how to achieve consistency per se but how to achieve principled consistency: decisions that are consistent because they reflect transparent, well-articulated normative reasoning, not because they result from the mechanical application of predetermined rules. The evidence demonstrates that voting should be restricted to exceptional circumstances, because it aggregates positions arithmetically while obscuring the quality of their justifications, substituting numerical weight for deliberative legitimacy (European Patients’ Academy on Therapeutic Innovation, 2021). Markham and Buchanan (2012) identify the grey areas of applied ethics as requiring enhanced deliberative practice: greater epistemic humility, broader perspective-taking, and more systematic engagement with dissenting positions. The results indicate that multidisciplinary composition expands the normative repertoire available for deliberation and reduces the risk of disciplinary tunnel vision (Mehta et al., 2023), but is insufficient without a deliberative culture that genuinely values heterogeneous perspectives.

### Theme 3: Structural and Communicative Standards of Opinion Quality

The thematic synthesis identified a convergent set of structural and communicative standards for quality opinions across the reviewed literature, despite the absence of a universally accepted format. Table 2 presents these standards organised across the three dimensions of the tripartite framework, specifying for each dimension its definition, quality indicators, and common deficiencies. Figure 1 illustrates the structural architecture of a quality opinion as a five-stage sequential process, showing the logical progression from identification through ethical evaluation to communicative documentation. Opinions that fail to meet these standards function as bureaucratic impositions rather than instruments of ethical dialogue, satisfying the legal function of the opinion while entirely failing its communicative and pedagogical functions (Serpico, 2024). The typology of opinions requires differentiated communicative standards: conditional opinions, which Martín-Arribas et al. (2012) document as the modal outcome (median of two protocol versions; median evaluation period of 13.5 days), should be framed as invitations to collaborative refinement, framed with specificity, normative justification, and constructive orientation toward resolution.

### Theme 4: Training, Consistency, and Institutional Learning

The reviewed literature consistently identifies member training as a necessary but insufficient condition for opinion quality. Mehta et al. (2023) document that lack of scientific and ethical competence among reviewers is a primary source of quality variation, and that systematic training in bioethics foundations, review procedures, and SOPs contributes to improving consistency. The American Society for Reproductive Medicine (2023) provides an exemplary institutional model of sophisticated opinion drafting in a complex clinical domain. However, training must be understood as a developmental process cultivating deliberative competence through sustained experience of applying principles in real deliberative contexts, not through the study of rules, consistent with the Aristotelian account of phronesis (Aristotle, 2009, Book II). Institutional mechanisms for deliberative learning, including systematic retrospective review of committee decisions for consistency and quality, structured reflection on difficult cases, inter-committee exchange, and formalised feedback from researchers, are therefore as important as formal training programmes. Chirico and Bramstedt (2022) propose that feedback mechanisms be formally expanded to include editorial and policymaking perspectives, transforming the committee from an isolated review body into a node in a broader network of ethical governance.

## Discussion

### Synthesis and Theoretical Integration

The thematic synthesis converges on a central argument that extends and integrates the existing literature: the quality of ethical opinions is determined by three interdependent dimensions that cannot be achieved independently. Normative grounding provides the substantive content of ethical evaluation: without mastery of foundational principles and applicable guidelines, no amount of deliberative sophistication or communicative care can compensate for the absence of the normative resources required to identify and assess ethical issues. Deliberative integrity provides the process conditions through which normative content is transformed into collective judgement: without communicative processes satisfying Habermasian norms of sincerity, inclusivity, and argumentative authority, individually held normative knowledge cannot become the shared practical wisdom of the committee as a whole. Communicative responsibility provides the documentary expression of that collective wisdom: without transparent, substantiated written opinions, the results of even the most rigorous deliberative processes remain institutionally invisible, unable to perform the legal, communicative, and pedagogical functions that constitute the committee's primary obligations.

### Interpretation in Relation to Existing Literature

These findings are consistent with and extend the existing literature in several respects. The finding that deliberative integrity is the primary determinant of opinion quality aligns with Friesen et al.'s (2019) argument for structured discretionary systems, extending it by identifying the specific communicative conditions that structured discretion must satisfy to achieve deliberative legitimacy, a gap that Friesen et al.'s (2019) analysis, which establishes the superiority of discretionary over algorithmic judgement without specifying the communicative norms distinguishing principled from arbitrary discretion. In contrast with the dominant approach in the literature addressing specific dimensions of REC governance without integration, the tripartite framework provides a comprehensive analytical structure connecting normative, deliberative, and communicative dimensions. This integration responds to the gap identified by Resnik (2021), who notes the absence of an adequate theoretical framework for understanding the specific epistemic and normative requirements of IRB decision-making. The framework extends Emanuel et al.'s (2004) eight-requirement model by adding an explicit communicative dimension, specifically the requirement that the committee's reasoning be documented with sufficient transparency to enable rational scrutiny, which the original framework does not address. The emphasis on *phronesis* as the irreducible core competence of ethical judgement diverges from approaches that privilege standardisation and compliance. The claim is not that standardisation lacks value: the evidence reviewed confirms that SOPs, training, and compositional requirements contribute measurably to quality; standardisation alone cannot achieve the contextual sensitivity required for ethical judgement to honour the particularity of human situations. These findings also engage with the research programme initiated by Tsan (2019) and Fernandez et al. (2019), which identifies the persistent absence of validated, outcome-oriented metrics for assessing IRB quality and effectiveness. The tripartite framework responds to this gap by providing a theoretically grounded architecture for quality assessment that moves beyond compliance-based indicators toward the deliberative and communicative dimensions that Tsan (2019) identifies as insufficiently measured and that Fernandez et al. (2019) argue are essential to determining whether IRBs actually achieve participant protection.

### Theoretical Contribution: Collective Phronesis as Institutional Capacity

A particularly noteworthy theoretical contribution concerns the concept of collective phronesis, understood as practical wisdom in an institutional rather than purely individual sense. Aristotle (2009) locates phronesis in the individual person of practical experience; the framework proposed here argues that committees, as deliberative institutions, develop a collective practical wisdom that is more than the sum of their members’ individual competencies, and is formed, maintained, and transmitted through the institutional mechanisms of learning, feedback, and retrospective reflection identified in Theme 4. This conceptual extension responds to a limitation in existing accounts (Beauchamp & Childress, 2019; Resnik, 2021): by specifying the institutional conditions under which practical wisdom becomes a collective rather than purely individual achievement, the framework provides a theoretically grounded account of what institutional investment in deliberative quality actually entails.

### Limitations

This study presents several limitations. As a narrative review, it does not follow systematic protocol registration and PRISMA-compliant procedures. The geographic concentration of the reviewed literature in North American and Western European contexts limits the generalisability of findings to the diverse institutional environments, including sub-Saharan Africa, South and East Asia, and Latin America, in which RECs operate under substantially different resource, regulatory, and cultural conditions. The absence of validated instruments for assessing opinion quality represents a further limitation: the quality standards proposed in the tripartite framework remain theoretically grounded but empirically unvalidated, pending the development of assessment instruments and their application in studies of REC practice.

## Conclusion

### Main Conclusions

This study demonstrates that the quality of ethical opinions produced by research ethics committees cannot be adequately understood through a lens of procedural compliance alone. The analysis responds to the three research questions by establishing: that sound opinions require the application of foundational principles as prima facie obligations rather than algorithmic rules (RQ1); that opinion quality is fundamentally determined by communicative transparency anchored in explicit normative justification (RQ2); and that institutional mechanisms for deliberative learning, and not merely formal training, are the primary supports of sustained quality over time (RQ3). These findings extend the work of Serpico (2024), Friesen et al. (2019), Resnik (2021), and Emanuel et al. (2004) by integrating their partial analyses into a unified tripartite framework connecting normative, deliberative, and communicative dimensions of REC practice.

### Theoretical Implications

Theoretically, this study advances the understanding of ethical deliberation in institutional contexts by demonstrating the complementarity of Habermasian communicative rationality and Aristotelian phronesis as foundational frameworks for committee-based ethical governance. By integrating these two philosophical traditions, the study demonstrates that communicative rationality provides the structural conditions for legitimate deliberation while phronesis provides the dispositional competencies required to exercise judgement wisely within those structural conditions. By introducing the concept of collective phronesis, understood as practical wisdom in an institutional capacity cultivated through experience, reflection, and deliberative learning, the framework contributes an original conceptual tool to the literature on research ethics governance that extends existing accounts (Beauchamp & Childress, 2019; Resnik, 2021) by specifying the institutional conditions under which practical wisdom becomes a collective rather than purely individual achievement. This study fosters debate about the appropriate institutional forms for ethical governance of research and anticipates broader relevance across governance bodies facing the challenge of principled judgement under conditions of irreducible complexity.

### Practical Implications

Practically, the tripartite framework provides committee members, administrators, and governance bodies with a theoretically grounded diagnostic and developmental instrument. Institutions should invest in structured opinion templates operationalising communicative transparency; in training programmes that cultivate deliberative competence through case-based exercises and structured reflection rather than procedural instruction alone; and in feedback mechanisms that transform researcher responses into evidence for continuous quality improvement. These recommendations extend the quality standards advocated by the WHO (2011) and European Patients’ Academy on Therapeutic Innovation (2021) by specifying the philosophical foundations that determine why these standards matter and what achieving them requires in practice. From a policy perspective, national research governance bodies should develop quality assessment frameworks for REC opinions that go beyond compliance checklists to evaluate the depth and transparency of normative reasoning, a development that Serpico's (2024) analysis of existing quality gaps identifies as both urgent and feasible.

### Educational Implications

The tripartite framework proposed in this study has direct educational implications for at least three distinct target audiences: members of research ethics committees, researchers and principal investigators, and institutional governance administrators and policymakers.

For committee members, the framework challenges training designs centred on procedural compliance and rule memorisation, arguing instead for developmental curricula grounded in case-based deliberation. Consistent with the Aristotelian account of *phronesis* (Aristotle, 2009, Book II), ethical competence is acquired through sustained practical experience in recognising, analysing, and resolving ethically complex situations, not through passive reception of normative content. Educational programmes should therefore incorporate structured case analyses of real committee deliberations, structured reflection on past opinions, and inter-committee exchange as mechanisms for cultivating the deliberative competence that formal instruction alone cannot produce. With respect to specific pedagogical methods suited to adult learners, the evidence supports two complementary approaches. First, experiential learning approaches – particularly problem-based learning and deliberative simulation exercises in which participants engage with authentic or reconstructed REC cases – are especially effective for developing the contextual sensitivity and practical judgement that *phronesis* requires, because they situate learning within the kinds of real decision-making environments in which competence must ultimately operate (Knowles et al., 2015). Second, structured reflection mechanisms, including facilitated post-meeting debriefs, peer feedback on opinion drafts, and systematic retrospective review of decisions, function as andragogical tools that transform experience into explicit learning, enabling committee members to progressively refine both their normative reasoning and their communicative practice. These two approaches are mutually reinforcing: experiential exercises generate the material for reflection, and structured reflection consolidates and extends the learning that experience initiates.

For researchers and principal investigators, the communicative transparency demanded of quality opinions has a corresponding educational counterpart: the capacity to design, justify, and present research protocols in ways that demonstrate active engagement with ethical principles rather than procedural compliance. Capili and Anastasi (2024) underscore the importance of researchers developing sufficient ethical literacy to engage productively with the review process, transforming the committee relationship from one of institutional gatekeeping into one of collaborative refinement. The integration of research ethics training into postgraduate and doctoral curricula should go beyond the declaratory presentation of normative frameworks to include practical exercises in protocol design, risk-benefit analysis, and informed consent documentation, using actual ethical opinions as pedagogical resources that render visible the normative reasoning of experienced committees.

For institutional governance administrators, the framework implies that investment in committee quality is simultaneously an educational investment. The institutional learning mechanisms identified in Theme 4, including systematic retrospective review of decisions, structured reflection on difficult cases, formalised feedback from researchers, and inter-committee exchange, constitute simultaneously quality assurance mechanisms and educational infrastructures through which collective phronesis is cultivated, transmitted, and renewed across successive cohorts of committee members.

### Best Practices

Building on the practical implications outlined in Section 6.3, the synthesis of the reviewed literature and the tripartite framework generates a set of operationalised best practices for research ethics committees, organised across the three interdependent dimensions of normative grounding, deliberative integrity, and communicative responsibility.

With respect to normative grounding, committees should: adopt the eight-requirement framework of Emanuel et al. (2004) as a structured evaluative instrument, ensuring that each dimension –including collaborative partnership – receives explicit and substantiated treatment in the written opinion; document, in cases of genuine conflict between prima facie principles, the reasoning through which competing principles were weighted rather than asserting conclusions without justification; and ensure that regulatory references are specific and traceable, identifying the applicable instrument, version, and provision with sufficient precision to allow researchers to locate the normative basis for committee requirements (Beauchamp & Childress, 2019; Council for International Organizations of Medical Sciences, 2016).

With respect to deliberative integrity, committees should: distribute all documentation at least five working days before scheduled meetings, ensuring that members enter deliberation with independent preparatory analysis rather than forming positions under social pressure; establish and enforce quorum requirements that reflect the multidisciplinary composition necessary for comprehensive ethical evaluation; restrict voting to exceptional circumstances and invest in facilitated consensus processes, recognising that numerical aggregation cannot substitute for argumentative legitimacy (European Patients’ Academy on Therapeutic Innovation, 2021); document deliberative reasoning with sufficient specificity to enable retrospective quality review; and implement structured mechanisms for identifying and managing conflicts of interest, including self-recusal and transparency requirements (WHO, 2011).

With respect to communicative responsibility, committees should: adopt standardised opinion templates operationalising the five-stage structural architecture illustrated in Figure 1; ensure that conditional opinions specify required modifications with reference to the protocol section requiring change, the normative basis for the requirement, and the standard against which revised submissions will be assessed, since the absence of any of these elements generates the arbitrariness documented by Serpico (2024); establish formal feedback mechanisms through which researcher responses to opinions are systematically collected and analysed as evidence for quality improvement; and invest in the systematic documentation and accessibility of institutional precedents, enabling new members to learn from the committee's accumulated deliberative history.

### Research Agenda

The findings and limitations of this study define a research agenda across three levels: methodological development, empirical investigation, and theoretical extension.

At the methodological level, the most urgent priority is the development and validation of instruments for the systematic assessment of ethical opinion quality. The tripartite framework provides the conceptual architecture for such instruments, but their operationalisation requires the translation of qualitative quality criteria into measurable indicators, their testing in diverse REC contexts, and their validation against independent assessments of opinion adequacy. Inter-rater reliability studies comparing expert assessments of opinion quality using the tripartite framework would constitute a necessary step toward establishing the empirical grounding that the framework currently lacks.

At the empirical level, cross-cultural comparative studies of deliberative practice and opinion standards across diverse REC systems, including the substantially under-researched systems operating in sub-Saharan Africa, South and East Asia, and Latin America, are needed to assess the generalisability of the framework beyond the North American and Western European contexts that dominate the available literature. Such studies would also illuminate the culturally differentiated ethical values that committees prioritise across different regional and institutional contexts, a dimension that the reviewer community has identified as insufficiently addressed in the existing governance literature. Cultural variation in the weighting of individual versus collective values, in community engagement practices, and in the normative authority accorded to different categories of expertise may produce substantially different deliberative architectures even within formally similar institutional structures; understanding these variations is a prerequisite for any future effort to develop cross-culturally valid quality assessment instruments. Longitudinal studies examining the effects of specific deliberative interventions, including structured case-based training, inter-committee exchange programmes, and feedback mechanism implementation, on measurable dimensions of opinion quality would provide the evidence base currently absent from the training literature. Such studies would respond directly to the challenge articulated by Tsan (2019) and Fernandez et al. (2019), who document the persistent absence of validated metrics for assessing IRB performance beyond regulatory compliance and argue for the development of outcome-oriented quality indicators grounded in participant protection rather than procedural conformity.

At the theoretical level, three directions merit priority. First, the concept of collective phronesis requires further theoretical development, particularly regarding the mechanisms through which individual practical wisdom is transformed into institutional capacity and the conditions under which institutional memory is maintained, transmitted, and renewed across changes in committee membership. Second, the growing intersection of research ethics with artificial intelligence and digital health research generates novel deliberative challenges, including questions of algorithmic accountability, data sovereignty, and the ethics of computational modelling, that require new normative frameworks extending beyond the principlist architecture designed for biomedical research involving human subjects. Third, the relationship between opinion quality and downstream research integrity outcomes requires empirical investigation: whether higher-quality ethical review, as defined by the tripartite framework, measurably reduces research misconduct, improves informed consent quality, or strengthens public trust in the research enterprise remains an open and consequential question.

### Final Considerations

The limitations and future research directions of this study are addressed in Sections 5.4 and 6.6 respectively. The ethical opinion, at its best, is not a bureaucratic output but a philosophical argument in action: the institutional form through which the aspiration to conduct science with integrity finds its most concrete and consequential expression. The capacity of research ethics committees to produce opinions that honour this aspiration is a collective achievement, formed through deliberation, sustained through learning, and renewed in each judgement that holds the imperative to advance knowledge accountable to the deeper imperative not to instrumentalise human beings.
